# The impact of chemoimmunotherapy on primary cold agglutinin disease and Waldenström macroglobulinemia-associated cold agglutinin syndrome

**DOI:** 10.3389/fmed.2026.1926130

**Published:** 2026-09-03

**Authors:** Kenichi Ito, Tomoko Kitagawa, Saya Motohashi, Kazuhiko Hirano, Naohiro Sekiguchi

**Affiliations:** 1 Hematology Division, National Hospital Organization Disaster Medical Center, Tachikawa, Tokyo, Japan; 2 Clinical Research Division, National Hospital Organization Disaster Medical Center, Tachikawa, Tokyo, Japan; 3 Laboratory and Pathology Division, National Hospital Organization Disaster Medical Center, Tachikawa, Tokyo, Japan

**Keywords:** chemoimmunotherapy, cold agglutinin disease, cold agglutinin syndrome, thrombotic events, Waldenström macroglobulinemia

## Abstract

**Background:**

Cold agglutinin-associated hemolysis (CAH) occurs in diverse clinical contexts, such as primary cold agglutinin disease (pCAD) and Waldenström macroglobulinemia-associated cold agglutinin syndrome (WM-CAS). The differentiation of these entities is often challenging, particularly in MYD88 L265P-negative cases. Since studies that examined the effects of chemoimmunotherapy (CIT) frequently predated routine molecular testing, it remains unclear whether disease classification impacts treatment response and durability.

**Purposes:**

This study aimed to evaluate the clinicopathological features, responses to CIT, long-term outcomes, and adverse events (AE), such as thrombotic events, among patients with CAH (pCAD and WM-CAS) and WM without CAS (WM-only).

**Methods:**

We retrospectively analyzed patients with pCAD, WM-CAS, and WM without CAS (WM-only) treated at a single center between April 2010 and November 2025. Diagnoses followed the revised fifth edition of the WHO Classification of Hematolymphoid Tumors. Clinicopathological features and outcomes were compared. Treatment responses were assessed using CAD-specific criteria based on hemoglobin and hemolysis markers. Exploratory pooled analyses combining pCAD and WM-CAS as the CAH group were also performed. Time to next treatment (TTNT) and overall survival (OS) were analyzed using Kaplan–Meier methods.

**Results:**

Ten patients had CAH (5 pCAD, 5 WM-CAS) and 29 had WM-only. In exploratory pooled comparisons, CAH cases showed higher lactate dehydrogenase and total bilirubin levels, whereas WM-only cases had higher serum IgM levels and greater bone marrow involvement. Rituximab-based CIT predominated as the first-line therapy for both pCAD and WM-CAS. Overall response rates were 100% in pCAD and 80% in WM-CAS. TTNT did not significantly differ between pCAD and WM-CAS, although this comparison was limited by the very small sample size. Elevated FDP levels were more frequent in CAH, while no overt thrombotic events or grade ≥3 infections were observed.

**Conclusion:**

In this small retrospective cohort, hematologic responses were observed after rituximab-based CIT in patients with both pCAD and WM-CAS. When hemolysis is the dominant clinical issue and strict disease classification is difficult, fixed-duration rituximab-based CIT should be further investigated. These hypothesis-generating findings warrant validation in larger studies.

## 1 Introduction

Cold agglutinins (CA) are autoantibodies (typically immunoglobulin [Ig] M-*κ*) that bind red blood cell antigens at low temperatures and trigger complement-mediated hemolysis (1). CA-associated hemolysis (CAH) occurs across diverse clinical contexts, such as lymphoid malignancies, infections, and autoimmune diseases (2–6). In addition, CAH may increase the incidence of thrombotic events as well as mortality (7–9).

In the fifth edition of the World Health Organization (WHO) Classification of Hematolymphoid Tumors, primary CA disease (pCAD) is defined as a neoplastic CA-producing condition arising from clonal B-cell proliferation that does not meet the diagnostic criteria for a specific B-cell lymphoma (10), whereas CAH associated with other underlying disorders, such as B-cell lymphomas, is classified as CA syndrome (CAS) (11). However, difficulties are associated with differentiating between pCAD and Waldenström macroglobulinemia-associated CAS (WM-CAS) in routine practice. Although *MYD88 L265P* mutation (*MYD88^L265P^*) testing and *IGHV4-34* usage are considered informative because *MYD88^L265P^* is detected in approximately 90% of WM cases (12), while IGHV4-34 usage is reported in the vast majority of CAD cases (13, 14), these molecular assessments are not always available, and diagnoses often rely on an integrated clinical and pathological evaluation (14). In clinical practice, strict categorization may not always be feasible.

Despite these diagnostic challenges, therapies targeting the underlying clonal disorder are widely used in clinical practice for patients with CAH. In WM-CAS, CAH is regarded as a symptomatic manifestation of WM (15) and is managed according to WM treatment recommendations (16); in contrast, pCAD may be managed with B-cell or plasma cell clone-directed therapy, including rituximab-based regimens (17–22), Bruton tyrosine kinase inhibitors (23), and anti-CD38 monoclonal antibodies, such as daratumumab (24), and/or complement-directed therapy, including sutimlimab (25).

However, many studies that evaluated chemoimmunotherapy (CIT) for CAD missed routine molecular testing and, thus, were unable to reliably distinguish pCAD from WM-CAS. Consequently, it remains unclear whether the disease classification affects the clinical presentation, responses to CIT, and outcomes of patients with CAH.

Therefore, we retrospectively analyzed patients with CAH (pCAD and WM-CAS) and WM without CAS (WM-only) to compare their clinicopathological features, responses to CIT, long-term outcomes, and adverse events (AE), including thrombotic events.

## 2 Methods

### 2.1 Patients and study design

We conducted a retrospective review of consecutive patients diagnosed with pCAD and WM (with or without CAS) who were treated at the National Hospital Organization Disaster Medical Center between April 2010 and November 2025. The diagnoses of pCAD and WM were established in accordance with the revised fifth edition of the WHO Classification of Hematolymphoid Tumors: Lymphoid Neoplasms (10, 26).

Cases of CAS secondary to infections or malignant disorders other than WM were excluded. These exclusions were based on a comprehensive evaluation, including clinical presentation, laboratory findings, pathological assessment, and *MYD88^L265P^* analysis.

### 2.2 Data collection

Clinical information was obtained from electronic medical records and included demographic characteristics (age and sex) and performance status (27). Hematological and biochemical parameters included the levels of hemoglobin, total bilirubin, lactate dehydrogenase (LDH), serum IgM, and fibrin degradation products (FDP). Additional clinical variables included CA titers; the presence of splenomegaly, lymphadenopathy, hyperviscosity syndrome, and B symptoms; and transfusion requirements. Bone marrow (BM)-based variables included cytogenetic abnormalities, the mutational status of *MYD88^L265P^* and *CXCR4^WHIM^*, and the percentages of lymphocytes and plasma cells in BM specimens. Treatment-related variables included treatment regimens, treatment responses, times to response, treatment-related AE graded according to the Common Terminology Criteria for Adverse Events (28), time to next treatment (TTNT), and overall survival (OS). TTNT was defined as the time from the initiation of first-line systemic therapy to the initiation of the next systemic therapy or death, whichever occurred first; in pCAD, next-line therapy was triggered by the need for additional treatment for recurrent or persistent hemolysis, in WM-only by the International Workshop on Waldenström’s Macroglobulinemia (IWWM) response criteria (29), and in WM-CAS by hemolysis and/or the IWWM response criteria. OS was defined as the time from the initiation of first-line systemic therapy to death. The data cutoff date was 30 November 2025.

### 2.3 Pathological and genetic analyses

BM aspirates and biopsy specimens were reviewed to classify CAH cases as pCAD or WM-CAS, in accordance with the revised fifth edition of the WHO Classification of Hematolymphoid Tumors: Lymphoid Neoplasms (10, 26).

All cases harboring *MYD88^L265P^* were classified as WM in this clinical context (26). In *MYD88^L265P^*-negative cases presenting with CAH, the disease classification was primarily based on BM biopsy findings, with BM aspiration findings used as supportive information.

Cases were classified as pCAD when BM biopsy showed intraparenchymal nodular infiltrates without overt tumor infiltration and were composed predominantly of small lymphoid cells with limited diffuse plasma cell involvement. In contrast, cases were classified as WM when BM biopsy revealed various infiltration patterns, such as diffuse involvement, with admixed populations of small B cells, lymphoplasmacytic cells, and plasma cells, consistent with previously reported pathological features (14). BM aspiration findings showing overt tumor cell infiltration were considered supportive of a diagnosis of WM.

A conventional chromosome banding analysis was performed on BM aspirates obtained at the time of the initial diagnosis. Genetic analyses were conducted using droplet digital PCR (ddPCR) with the QX200 AutoDG Droplet Digital PCR System (Bio-Rad). Commercial ddPCR mutation detection assays (Bio-Rad) were used to identify *MYD88^L265P^* and *CXCR4* mutations, such as *T318* frameshift, *S338* nonsense, and *S338* frameshift variants, as previously described (30).

### 2.4 Treatment and response assessment

Initial treatment regimens were selected at the discretion of the treating physicians according to contemporaneous clinical practice.

Treatment responses in patients with CAH (pCAD and WM-CAS) were assessed using CAH response criteria, as applied in previous clinical studies of ibrutinib and daratumumab (23, 24). In WM-CAS, tumor responses were also assessed using the IWWM response criteria (29), and responses in WM-only were assessed using the IWWM criteria. According to the CAH response criteria (23, 24), a CAH-complete response (CAH-CR) was defined as a hemoglobin level >12 g/dL, resolution of the clinical signs of hemolysis, independence from red blood cell transfusion, and the normalization of total bilirubin and LDH levels. A CAH-partial response (CAH-PR) was defined as a hemoglobin level of 10–12 g/dL or an increase of ≥2 g/dL from baseline without recent transfusion, accompanied by improvements in hemolytic markers without complete normalization. Patients who did not meet these criteria were classified as having no response. The overall response rate (ORR) was calculated as the percentage of patients achieving CAH-CR or CAH-PR.

### 2.5 Statistical analysis

**Figure 1:**
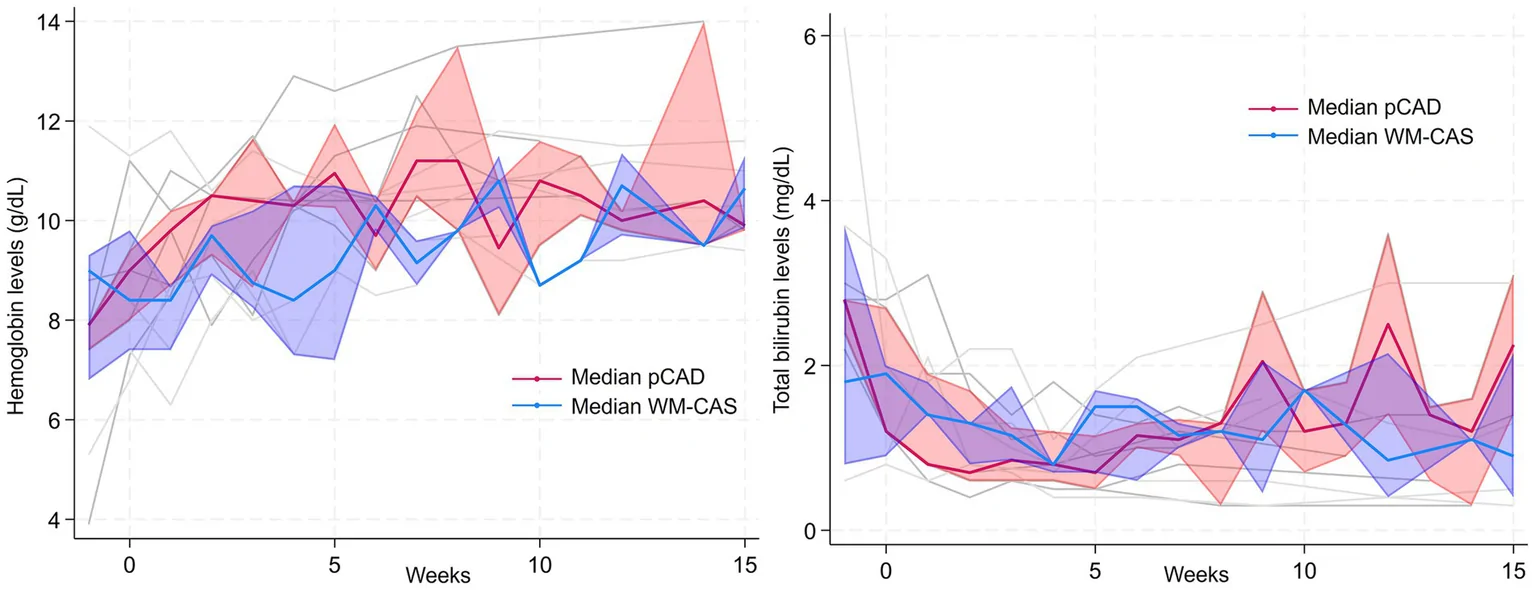
Chronological changes in laboratory findings after treatment. **(a, b)** Hemoglobin and **(b)** total bilirubin over time. The x-axis indicates weeks, with week 0 marking treatment initiation and negative weeks indicating pretreatment measurements. Red and blue lines show median values for primary cold agglutinin disease (pCAD) and Waldenström macroglobulinemia-associated cold agglutinin syndrome (WM-CAS), respectively, with the shaded areas indicating the 95% confidence intervals. Light- and dark-gray lines represent individual patient trajectories for pCAD and WM-CAS.

In addition to disease-specific analyses, pCAD and WM-CAS were pooled as the CAH group for exploratory comparisons with WM-only. This approach was based on the shared clinical phenotype of cold agglutinin-associated hemolysis and was not intended to imply biological equivalence between pCAD and WM-CAS. TTNT and OS were analyzed using the Kaplan–Meier method, and differences between groups were assessed using the log-rank test. Comparisons of categorical variables between two groups were performed using Fisher’s exact test, and comparisons of continuous variables were conducted using the Wilcoxon rank-sum test. In Figure 1, 95% confidence intervals around the median trajectories were estimated using bootstrap resampling. All statistical analyses were performed using Stata version 18 (StataCorp [LLC], College Station, TX, USA).

## 3 Results

### 3.1 Patient characteristics

Five patients had pCAD, five had WM-CAS, and 29 had WM-only. Baseline characteristics are summarized in Table 1. Characteristics stratified by the presence or absence of CAH are shown in Supplementary Table S1.

**Table 1:** Patient characteristics according to disease phenotype (primary CAD, WM-CAS, and WM without CAS).

Characteristic	(1) primary CAD (*N* = 5)	(2) WM with CAS (*N* = 5)	(3) WM without CAS (*N* = 29)
Median age, years (range)	68 (48–85)	71 (64–80)	71.5 (49–85)
Sex (M/F), *n*	2/3	2/3	25/4
PS > 1, *n* (%)	3/5 (60)	2/5 (40)	7/29 (24)
Hb < 11.5 g/dL, *n* (%)	5/5 (100)	4/5 (80)	22/29 (76)
Plt < 100 × 10^9^ /L, *n* (%)	0/5	1/5 (20)	2/29 (7)
β2MG > 3 mg/L, *n* (%)	0/3	3/5 (60)	18/27 (67)
Serum ALB < 3.5 g/dL, *n* (%)	1/5 (20)	1/5 (20)	19/29 (66)
LDH > 250 IU/L, *n* (%)	3/5 (60)	1/5 (20)	1/28 (4)
FDP > ULN μg/mL, *n* (%)	3/5 (60)	2/5 (40)	3/29 (10)
Median serum IgM, mg/dL (range)	232 (162–1,077)	697 (327–6,810)	3,191 (341–7,682)
Median M-protein, g/dL (range)	1 (0.8–1.3)	1.4 (0.8–3.9)	3 (1.1–6.6)
*MYD88 L265P* mutation, *n* (%)	0/4	1/4 (25)	27/29 (93)
*CXCR4* mutation, *n* (%)	0/4	0/4	3/29 (10)
Deletion chromosome 17p, *n* (%)	0/5	0/4	2/25 (8)
Complex karyotype, *n* (%)	0/5	1/4 (25)	4/29 (14)
Hyperviscosity, *n* (%)	0/5	0/5	12/29 (41)
B-symptom, *n* (%)	0/5	0/5	8/29 (28)
Splenomegaly, *n* (%)	4/5 (80)	2/5 (40)	7/29 (24)
Lymphadenopathy, *n* (%)	0/5	1/5 (20)	11/29 (38)
Median total neoplastic cells in BM, % (range)	19 (8–33)	30.7 (18.8–62)	47.1 (35.1–86.7)

CAD, cold agglutinin disease; WM, Waldenström macroglobulinemia; CAS, cold agglutinin syndrome; PS, performance status; Hb, hemoglobin; Plt, platelets; β2MG, β2-microglobulin; ALB, albumin; LDH, lactate dehydrogenase; FDP, fibrin/fibrinogen degradation products; IgM, immunoglobulin M; BM, bone marrow.

Median serum IgM levels were 232 mg/dL in pCAD, 697 mg/dL in WM-CAS, and 3,191 mg/dL in WM-only. FDP elevation was more frequent in pCAD and WM-CAS than in WM-only, being observed in 3/5 patients (60%), 2/5 patients (40%), and 3/29 patients (10%), respectively. In contrast, MYD88 L265P was detected predominantly in WM-only, being absent in pCAD, present in 1/4 WM-CAS patients (25%), and detected in 27/29 WM-only patients (93%).

### 3.2 Individual characteristics of CAH

The individual characteristics of the 10 CAH cases are shown in Table 2. The underlying diagnoses were pCAD in five patients, which had been previously classified as symptomatic pCAD (19), and WM-CAS in five patients.

**Table 2:** Individual patient characteristics with cold agglutinin-associated hemolysis.

Case	Underlying disease	Age/sex	Hb (g/dL)	T-bil (mg/dL)	IgM (mg/dL)	CA titer	Splenomegaly	Lymph-adenopathy	Isotype of the M protein	G-banding	MYD88 mutation	CXCR4 mutation	Bone marrow involvement pattern	Lymphocytes and plasmacytes in BM	RBC transfusion before therapy (U/month)
1	pCAD	61/M	9	3	1,077	4,096	+	−	IgM-κ	normal karyotype	wild type	wild type	nodular	20%	8
2	pCAD	68/M	7.5	2.8	191	256	+	−	IgM-*κ*	47, XY, +X [20/20]	wild type	wild type	nodular	18%	4
3	pCAD	48/F	3.9	2.2	888	512	+	−	IgM-κ IgG-κ	normal karyotype	wild type	wild type	No aggregates	4.4%	6
4	pCAD	74/F	7.9	2.8	232	2,048	−	−	IgM-κ	normal karyotype	wild type	wild type	No aggregates	10.4%	4
5	pCAD	85/F	7.9	1.2	162	512	+	−	IgM-κ	normal karyotype	NA	NA	nodular	11%	0
6	WM	80/M	9.3	0.8	1,340	64	−	−	IgM-κ	normal karyotype	Mutated	wild type	nodular	36%	0
7	WM	76/F	5.3	1.8	6,810	2,048	+	−	IgM-κ IgA-λ	49, XX, +add(1)(p11), +18, +20 [2/20]	wild type	wild type	diffuse	62%	8
8	WM	64/M	11.9	3.7	327	1,024	+	−	IgM-κ IgG-κ	normal karyotype	wild type	wild type	nodular	31%	0
9	WM	68/F	9	0.6	627	256	−	−	IgM-κ	normal karyotype	wild type	wild type	diffuse	28%	0
10	WM	71/M	6.8	6.1	697	4,096	−	+	IgM	NA	NA	NA	nodular	19%	8
Median (range)		69.5 (48–85)	7.9 (3.9–11.9)	2.5 (0.6–6.1)	662 (162–6,810)	768 (64–4,096)								19.5% (4.4–62)	4 (0–8)

pCAD, primary cold agglutinin disease; WM, Waldenström macroglobulinemia; M, male; F, female; Hb, hemoglobin; T-bil, total bilirubin; IgM, immunoglobulin M; CA, cold agglutinin; BM, bone marrow; RBC, red blood cell; U, units; LPL NA, not applicable.

In the pCAD group, *MYD88^L265P^* analysis was performed in four patients, and none had the mutation. Regarding marrow infiltration patterns, three patients (Cases 1, 2, and 5) showed a nodular pattern, whereas no overt neoplastic infiltration was identified in the remaining cases.

In the WM-CAS group, *MYD88^L265P^* analysis was available for four patients, and it was positive in 1/4 (Case 6). Case 10 had lymphadenopathy. Cases 7 and 9 showed diffuse BM infiltration on biopsy. Case 8 showed a nodular pattern on biopsy; however, 31% tumor cell infiltration was confirmed in a BM aspirate, and the case was diagnosed as LPL.

### 3.3 Treatment regimens

In all patients with WM-CAS, treatment was initiated for clinically significant anemia. The relative contributions of cold agglutinin-associated hemolysis and the underlying WM to the anemia could not be clearly distinguished.

Treatment regimens were distributed as follows. In the pCAD group, four patients received rituximab, cyclophosphamide, doxorubicin, vincristine, and prednisone (R-CHOP), and one received bendamustine plus rituximab (BR) (22). In the WM-CAS group, R-CHOP was administered to five patients (29). In the WM-only group, R-CHOP was the most frequently used regimen (*n* = 20), followed by BTK inhibitors (*n* = 5), BR (*n* = 1), rituximab monotherapy (*n* = 1), bortezomib-based regimens (*n* = 1), and other treatments (*n* = 1).

### 3.4 Response of hemolytic anemia assessed by CAH response criteria

Figure 1A shows chronological changes in hemoglobin and total bilirubin levels, reflecting the treatment responses of hemolytic anemia in patients with pCAD and WM-CAS.

In the pCAD group, all patients (5/5) achieved at least CAH-PR (ORR 100%). The median time to CAH-PR was 3 weeks (range, 1–24). In addition, 4/5 patients achieved CAH-CR, with a median time to CAH-CR of 21.5 weeks (range: 4–41).

In the WM-CAS group, 4/5 patients achieved at least CAH-PR, yielding an ORR of 80%. Case 8 did not meet the criteria for PR according to the CAH response criteria because the baseline hemoglobin level was already above 10 g/dL and the increase from baseline was less than 2 g/dL. Case 6, the only patient with confirmed MYD88 L265P-mutated WM-CAS, achieved a CAH-PR but had stable disease according to the IWWM criteria and was lost to follow-up after 2 months. The median time to CAH-PR was 5 weeks (range, 2–6). In addition, 2/5 patients subsequently achieved CAH-CR, with a median time to CAH-CR of 29.5 weeks (range, 21–38).

### 3.5 Tumor response assessed by IWWM response criteria

Based on IWWM response criteria (29), the best overall responses were CR (*n* = 2), PR (*n* = 2), and SD (*n* = 1) in the WM-CAS group, and CR (*n* = 4), VGPR (*n* = 8), PR (*n* = 10), and SD (*n* = 7) in the WM-only group.

### 3.6 Survival outcomes

**Figure 2:**
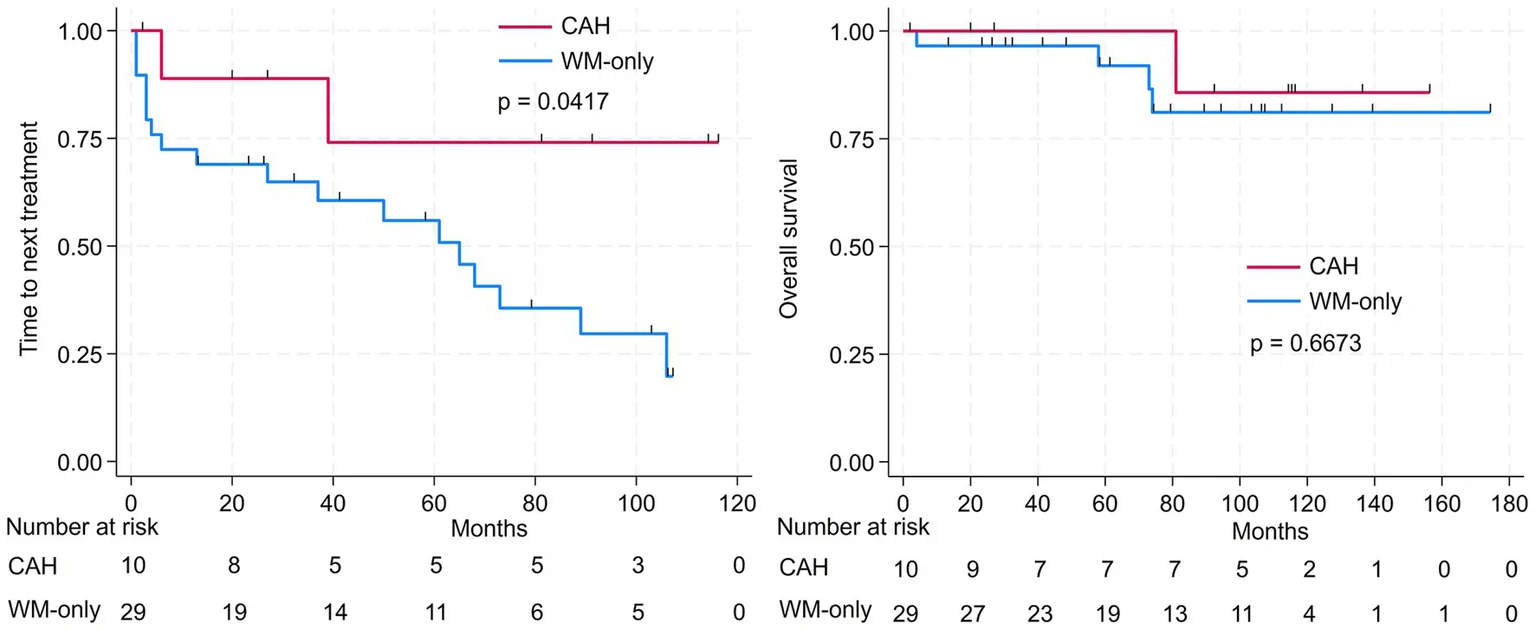
Survival analyses. **(a)** Time to next treatment (TTNT) and **(b)** overall survival (OS). The *x*-axis shows time in months. Kaplan–Meier curves compare patients with cold agglutinin–associated hemolysis (CAH; primary cold agglutinin disease and Waldenström macroglobulinemia–associated cold agglutinin syndrome) (red) versus patients with Waldenström macroglobulinemia without cold agglutinin syndrome (WM-only) (blue). Because this analysis pooled biologically and clinically distinct pCAD and WM-CAS and included only a small number of patients, it was considered exploratory, and the results should be interpreted with caution.

Exploratory pooled comparisons of TTNT and OS in patients with CAH (pCAD and WM-CAS) versus WM-only are shown in Figures 2a,b, respectively. The median follow-up was 81 months. Median TTNT was not reached in the CAH group and was 65 months in the WM-only group, with a significant difference between the groups (Log-rank *p* = 0.0417). This finding was based on an exploratory pooled analysis combining biologically and clinically distinct pCAD and WM-CAS and should therefore be interpreted with caution.

**Figure 3:**
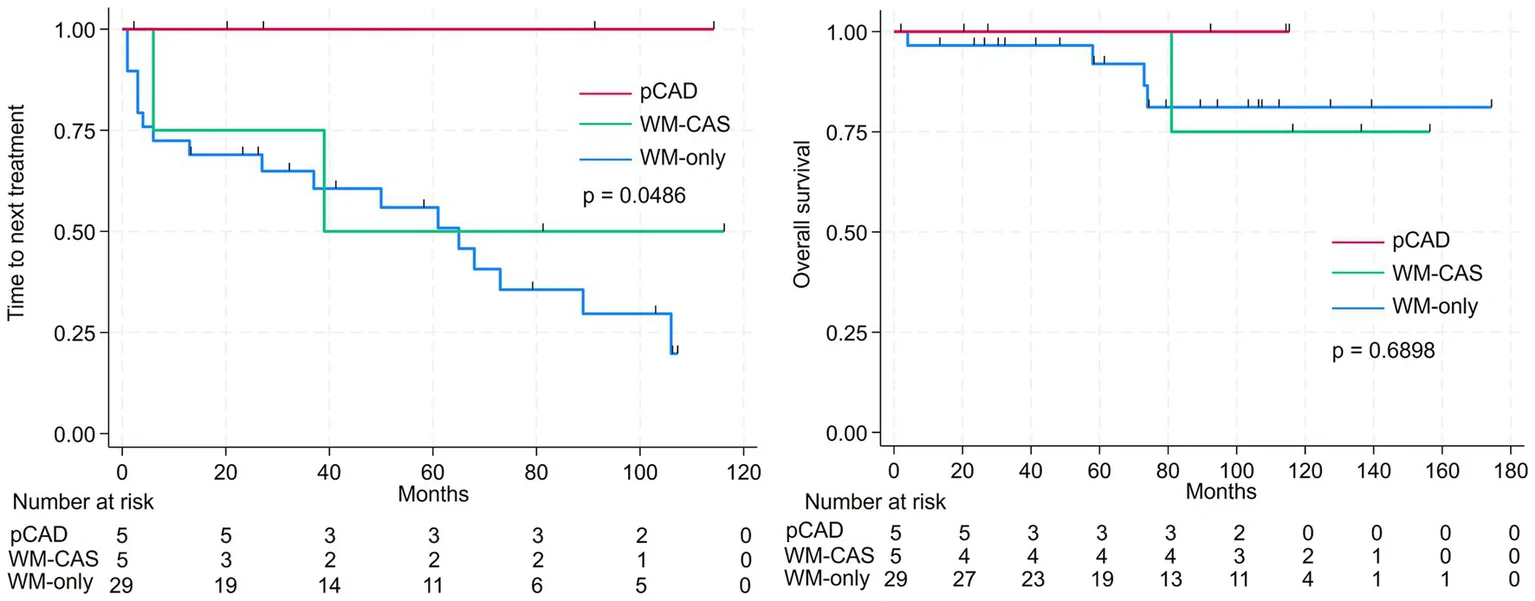
Survival analyses. **(a, b)** Time to next treatment and **(b)** overall survival. The x-axis indicates months. Red, green, and blue curves represent primary cold agglutinin disease (pCAD), Waldenström macroglobulinemia-associated cold agglutinin syndrome (WM-CAS), and Waldenström macroglobulinemia without cold agglutinin syndrome (WM-only), respectively.

TTNT is shown in Figure 3a and OS in Figure 3b for pCAD, WM-CAS, and WM-only. Median TTNT was not reached in the pCAD group, was 39 months in the WM-CAS group, and was 65 months in the WM-only group. TTNT did not significantly differ between pCAD and WM-CAS (log-rank *p* = 0.1343). Given the very small number of patients in each group, the absence of a statistically significant difference should not be interpreted as evidence of similar treatment durability between pCAD and WM-CAS.

No deaths occurred in the pCAD group. In the WM-CAS group, one patient died by suicide. In the WM-only group, three patients died of disease progression and one died of treatment-related complications during the follow-up. Median OS was not reached in any group.

Patient-level CAH responses, TTNT, and subsequent treatments are summarized in Supplementary Table S3.

### 3.7 Exploratory analysis according to MYD88 L265P status

An exploratory descriptive analysis according to MYD88 L265P status, irrespective of pathological classification, is presented in Supplementary Table S2 and Supplementary Figure S1. Baseline characteristics and CAH ORR are summarized in Supplementary Table S2, whereas TTNT and OS are shown in Supplementary Figures S1a, S1b, respectively. As only one patient was MYD88 L265P-positive, no statistical testing was performed.

### 3.8 Adverse events

Grade 3–4 neutropenia occurred in four pCAD patients and two WM-CAS patients. Two pCAD patients developed grade 2 infections. No thrombotic events occurred among the 10 CAH cases during follow-up.

## 4 Discussion

The accurate differentiation of pCAD from WM-CAS remains challenging in routine clinical practice; however, previous studies have indicated the potential of the *MYD88^L265P^* status, Ig gene usage (e.g., IGHV4-34), and BM infiltration patterns to support the disease classification (14). Somatic hypermutation of the Ig genes is observed in all cases of CAD (13), and *MYD88^L265P^* is typically absent in CAD (14). In the present study, Ig gene repertoire analysis could not be performed because it was not readily available in Japan. Similarly, many previous reports did not routinely include IGHV analysis (17–22). In contrast, *MYD88^L265P^* was negative in all pCAD cases tested (0/4) and was only detected in one of the WM-CAS cases (1/4). Importantly, *MYD88* wild-type (*MYD88^WT^*) cases have been reported even among WM-CAS; in a multicenter retrospective series on cold autoimmune hemolytic anemia, two of five WM-associated cases were *MYD88^WT^* (23). At the same time, available evidence remains insufficient to define the true frequency of *MYD88^L265P^* positivity or IGHV4-34 usage specifically in WM-CAS, and the BM pathology may be non-discriminatory when the tumor burden is low; therefore, a misclassification, particularly among *MYD88^WT^* cases, cannot be fully excluded (31, 32). Due to these diagnostic limitations, an important practical question is whether pCAD and WM-CAS show marked differences in treatment responses and durability.

Although pCAD and WM-CAS represent distinct disease concepts and therapeutic paradigms differ in principle, treatment selection in routine clinical practice often overlaps. This may reflect the fact that clinically significant hemolysis is a shared driver of interventions in both pCAD and WM-CAS. In WM-CAS, CAH is a symptomatic manifestation of WM (16) and typically prompts WM-directed systemic therapy according to established algorithms (15). On the other hand, in pCAD, treatment options include complement inhibition and clone-directed therapy. Complement inhibition may rapidly control hemolysis, but generally requires ongoing, costly treatment (22, 25, 33, 34) and does not target the underlying B-cell clone. Before the advent of complement inhibition, treatment was therefore largely based on clone-directed approaches, including rituximab monotherapy and rituximab-based CIT. Berentsen et al. reported a median response duration of 11 months with rituximab monotherapy, suggesting limited durability (17). In contrast, responses appear to be more durable with fixed-duration rituximab-based CIT (18, 19, 22); in a Nordic prospective multicenter study on BR in CAD, the median observed response duration was 32 months (19). During the study period, complement inhibitors were not yet available, and R-CHOP was one of the major CIT options available for untreated WM in Japan (29). In this context, CIT predominated as the first-line therapy in our cohort, including both pCAD and WM-CAS, and no statistically significant difference in TTNT was detected between the groups; TTNT was not reached in pCAD and was 39 months in WM-CAS (Log-rank *p* = 0.1343). However, this comparison should be interpreted cautiously because the criteria for initiating subsequent therapy differed between the groups. Therefore, the absence of a statistically significant difference should not be interpreted as evidence of comparable treatment durability between pCAD and WM-CAS. The overall response rate was 100% in the pCAD group and 80% in the WM-CAS group; however, these response rates should be interpreted with caution because of the very small sample size. They should also be interpreted in the context of historical treatment practice, as R-CHOP predominated in this cohort and contemporary management increasingly incorporates BR, BTK inhibitors, and complement-directed therapies, limiting the applicability of these findings to current practice. The longer TTNT in the pooled CAH group should be interpreted cautiously. Although this group had higher baseline hemolytic markers than the WM-only group, this study was not designed to assess whether these markers were associated with TTNT. Given the small sample size, the apparent association may be coincidental. The pooled analysis was based on the shared clinical presentation of hemolysis and was not intended to imply biological equivalence between pCAD and WM-CAS. As pCAD and WM-CAS are biologically and clinically distinct disease entities, pooling them may obscure disease-specific differences in treatment response and durability. The TTNT difference may partly reflect the inclusion of pCAD, in which median TTNT was not reached. It may also reflect differences among the groups in the indications for subsequent treatment. Despite these limitations, the hematologic responses observed in both groups suggest that fixed-duration rituximab-based CIT may warrant further investigation when hemolysis is the dominant clinical issue and strict disease classification is difficult. However, the present study cannot establish the comparative efficacy or optimal role of CIT in either pCAD or WM-CAS.

Patients with CAH are known to have an increased risk of thromboembolic events, likely related to complement activation and hemolysis-associated hypercoagulability (7–9). Furthermore, thromboembolic events may occur regardless of the season (9). In an IgM monoclonal gammopathy-associated CAD/cryoglobulinemia series (*n* = 11), transfusion-independent hemoglobin improvement (≥2 g/dL) was achieved in 63.6% of patients; however, mortality remained high (63.6%), potentially related to advanced disease at presentation, associated with a median diagnostic delay of 12 months (6). Accordingly, CIT for CAH requires careful monitoring for thromboembolic and infectious complications. In our cohort, elevated FDP at presentation was more frequent in CAH cases than in WM-only cases, which is consistent with a prothrombotic tendency, whereas no overt thrombotic events were observed. Neutropenia occurred with rituximab-based CIT; however, no grade ≥3 infections were documented.

This study is limited by its single-center retrospective design and small sample size, which restricts statistical power and precision for AE and event-time estimates. In addition, some exploratory analyses pooled pCAD and WM-CAS despite their distinct biological and clinical characteristics, which may have obscured disease-specific differences and further limited the generalizability of the findings. Furthermore, the criteria for initiating subsequent therapy differed among the disease groups, limiting the interpretability of direct TTNT comparisons. Molecular stratification was incomplete because an IGHV gene usage analysis (including IGHV4-34) was not performed and *MYD88^L265P^* testing was missing in one case each of pCAD and WM-CAS. Consequently, residual uncertainty in disease classification remains, and misclassification, particularly among MYD88L265P-negative or untested cases, cannot be fully excluded.

In conclusion, in this small retrospective single-center cohort, hematologic responses were observed after rituximab-based CIT in patients with both pCAD and WM-CAS. Although diagnostic overlap remains, particularly among MYD88L265P-negative cases, these findings suggest that fixed-duration rituximab-based CIT should be further investigated when CAH is clinically dominant and strict disease classification is difficult. These hypothesis-generating findings warrant validation in larger studies.

## Data Availability

The raw data supporting the conclusions of this article will be made available by the authors, without undue reservation.
